# A computational-experimental strategy identifies K-7174 against African swine fever

**DOI:** 10.52601/bpr.2025.250054

**Published:** 2026-10-31

**Authors:** Bozhang Sun, Fei Xi, Zhirui Liu, Huanan Liu, Haiyang Song, Yifei Zhao, Zixiang Zhu, Haixue Zheng, Guoliang Zhu

**Affiliations:** 1 State Key Laboratory for Animal Disease Control and Prevention, College of Veterinary Medicine, Lanzhou University, Lanzhou Veterinary Research Institute, Chinese Academy of Agricultural Sciences, Lanzhou 730000, China; 2 Gansu Province Research Center for Basic Disciplines of Pathogen Biology, Lanzhou 730046, China

**Keywords:** African swine fever virus, RNA polymerase, Virtual screening, Antiviral inhibitor, Molecular docking

## Abstract

African swine fever (ASF), caused by the African swine fever virus (ASFV), poses a catastrophic threat to global swine production due to the absence of effective vaccines or therapeutics. Targeting the virally encoded RNA polymerase (RNAP), which is essential for viral transcription, offers a promising antiviral strategy. Capitalizing on our previously resolved high-resolution structure of ASFV RNAP, we conducted a rigorous structure-based virtual screening of two compound libraries (T001 and LF1000) to identify selective inhibitors. From 100 candidates validated *in vitro*, nine compounds identified at the genome level by qPCR demonstrated >98% inhibition of ASFV replication in porcine alveolar macrophages (PAMs). The lead compound, K-7174 — a repurposed agent known to modulate proteasome and GATA activity — exhibited potent, dose-dependent antiviral efficacy (*IC*_50_ = 2.502 µmol/L), minimal cytotoxicity, and high binding affinity for ASFV RNAP. Molecular docking simulations indicated potential interactions with key catalytic residues, including Glu1051 and Ala456, proposing a mechanism that may involve active-site disruption. However, definitive confirmation of this binding pose requires further experimental validation through high-resolution structural methods such as cryo-EM. Our study not only nominates K-7174 as a promising anti-ASFV candidate but also establishes an integrated computational–experimental framework for accelerating the discovery of antiviral agents targeting viral polymerases.

## INTRODUCTION

African swine fever (ASF) is a highly contagious and frequently fatal hemorrhagic disease affecting domestic pigs and wild boars, with mortality rates approaching 100% (Blome *et al.* 2012; Gabriel *et al.* 2011). The causative agent, African swine fever virus (ASFV), is a large, enveloped double-stranded DNA virus and the sole member of the *Asfarviridae* family (Wang *et al.* 2021). Since its initial identification in Kenya in the early 20th century (Eustace Montgomery 1921), ASF has undergone extensive geographical spread. Severe outbreaks in Europe and Asia, particularly since 2018 in China, have caused considerable economic losses worldwide (Qu *et al.* 2022; Tran *et al.* 2022). The ASFV genome, ranging from 170 to 193 kb, encodes 150–200 proteins, including approximately 68 structural and over 100 non-structural proteins (Craig *et al.* 2021; Yang *et al.* 2023). Owing to the size and complexity of the virus, the structure and function of many key proteins involved in its life cycle remain poorly characterized, significantly impeding the development of effective vaccines and antiviral drugs.

As a nucleocytoplasmic large DNA virus (NCLDV), ASFV shares similarities with vaccinia and monkeypox viruses in encoding its own eukaryotic-like RNA polymerase (RNAP), allowing it to carry out transcription autonomously, independent of the host transcriptional apparatus (Cackett *et al.* 2020a, b). This RNAP is central to the viral life cycle and represents a highly promising target for antiviral drug development. The structural distinctions between viral RNAP and host Pol II provide a sufficient basis for the development of selective antiviral inhibitors. For instance, remdesivir — a prodrug of an adenosine analog — acts by incorporating into the elongating RNA chain and causing delayed termination (Kokic *et al.* 2021; Yin *et al.* 2020). It has been approved for treating COVID-19 and demonstrates broad *in vitro* activity against viruses such as Ebola and human coronaviruses (Beigel *et al.* 2020; Sheahan *et al.* 2017). Similarly, the recent global emergence of the human monkeypox virus (MPXV), with over 50,000 cases reported across 99 countries and territories (Salcedo and Madariaga 2023), has accelerated efforts to identify novel antiviral agents. Several inhibitors targeting MPXV transcription have been identified with potential broad-spectrum activity against poxviruses (Silhan *et al.* 2023; Wang *et al.* 2023).

Structure-based virtual screening has become an indispensable tool in early drug discovery, enabling rapid and cost-effective identification of high-affinity ligands by leveraging target protein structures, molecular docking, and binding affinity predictions, followed by experimental validation. This approach has yielded successful antiviral agents across diverse virus families, such as virtual screening of inhibitors targeting the RNA polymerase of MPXV (Zhao *et al.* 2025). For MPXV, homology modeling based on vaccinia virus RNAP has facilitated the proposal of candidate inhibitors, illustrating the utility of computational strategies against complex viral targets (Altayb 2022).

Recent structural advances from our group and others have resolved the architecture of the ASFV RNAP core and elongation complexes, unveiling the virus-specific transcription factor M1249L and providing high-resolution structural insights (Pilotto *et al.* 2024; Zhao *et al.* 2024; Zhu *et al.* 2025). Importantly, ASFV RNAP is resistant to classical inhibitors of bacterial (*e*.*g*., rifampicin) and eukaryotic (*e*.*g*., α-amanitin) RNA polymerases (Caeiro and Costa 1989; Kuznar *et al.* 1980), highlighting the potential for developing selective ASFV-specific inhibitors. Capitalizing on these findings, we employed a high-resolution structural model of ASFV RNAP (PDB: 8XX4) (Zhu *et al.* 2025) and host *Sus scrofa* RNA polymerase II (Pol II) (PDB: 7NVS) (Aibara *et al.* 2021) to conduct a targeted virtual screening of two commercial compound libraries: the T001 library of known active molecules and natural products, and the LF1000 diversity core library, which features extensive structural diversity and high-quality, drug-like and lead-like compounds. Through molecular docking and binding affinity analysis, we identified 396 candidate compounds that target ASFV RNAP without binding to *Sus scrofa* Pol II. The top 50 candidates from each library were evaluated using cell-based antiviral assays, leading to the identification of nine hits with anti-ASFV activity. Among these, K-7174 — a compound previously implicated in inhibition of cell adhesion, suppression of VCAM-1 expression, and antitumor activity (Imagawa *et al.* 2003; Kikuchi *et al.* 2013; Umetani *et al.* 2000) — was selected due to its favorable cytotoxicity profile for further mechanistic investigation. In this study, we demonstrate for the first time that K-7174 exhibits potent inhibition of ASFV replication *in vitro* with low cytotoxicity, highlighting its promise as a lead compound and establishing a rational structure-based framework for anti-ASFV drug discovery.

## RESULTS

### Structural comparison and binding site characterization of ASFV and *Sus scrofa* RNA polymerases

Eukaryotic Pol II is a multi-subunit complex essential for transcriptional regulation. The ASFV RNAP shares conserved core subunits with host Pol II but adopts a unique minimal octameric architecture. To identify selective inhibitors targeting the viral polymerase, we performed a structural comparison between ASFV RNAP (PDB: 8XX4) and *Sus scrofa* Pol II (PDB: 7NVS). Structural alignment revealed a highly conserved magnesium ion-binding site, which is critical for catalytic nucleotide incorporation, in both polymerases (Fig. 1A). Nonetheless, key structural differences were observed in the surrounding pocket, including variations in architecture and amino acid composition, which provide a structural basis for inhibitor screening. Using Molecular Operating Environment (MOE), we predicted druggable binding pockets adjacent to this Mg^2^^+^ site in both structures (Figs. 1B and 1C). These regions were selected for subsequent virtual screening. Both structures were optimized using Schrödinger’s Protein Preparation Wizard (Madhavi Sastry *et al.* 2013), including protonation, hydrogen addition, and energy minimization with the OPLS4 force field, to ensure high structural fidelity.

**Figure 1:**
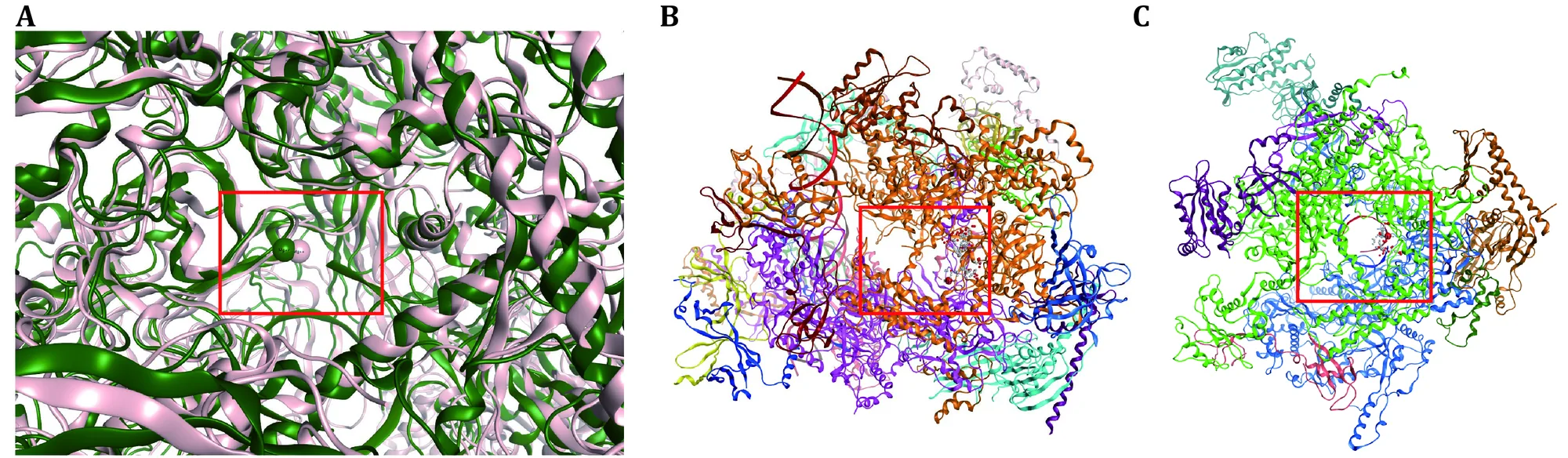
Structural comparison and binding site prediction of ASFV and *Sus scrofa* RNA polymerases. **A** Structural superposition of ASFV RNAP (dark green; PDB: 8XX4) and *Sus scrofa* Pol II (pink; PDB: 7NVS). The red box indicates the conserved Mg^2+^-binding site. **B** Predicted druggable pockets (red and gray spheres) near the Mg^2+^ site in *Sus scrofa* Pol II, identified using MOE. **C** Analogous predicted binding pockets in ASFV RNAP, used for virtual screening

### Structure-based virtual screening for selective ASFV RNAP inhibitors

We conducted a multi-step virtual screening campaign using two distinct compound libraries: a curated library of known bioactive compounds (T001) and a diverse synthetic library (LF1000). Ligand preprocessing was performed through generating stereoisomers, tautomers, and protonation states at pH 7.0 ± 2.0, along with conformational expansion (Greenwood *et al.* 2010; Shelley *et al.* 2007). Initial docking was performed against the prepared ASFV RNAP structure (8XX4) (Jacobson *et al.* 2004). Compounds exhibiting binding affinities ≤−6.0 kcal/mol were advanced to a counter-screen against the host *Sus scrofa* Pol II structure (7NVS) to filter for selective viral inhibitors (Fig. 2A).

**Figure 2:**
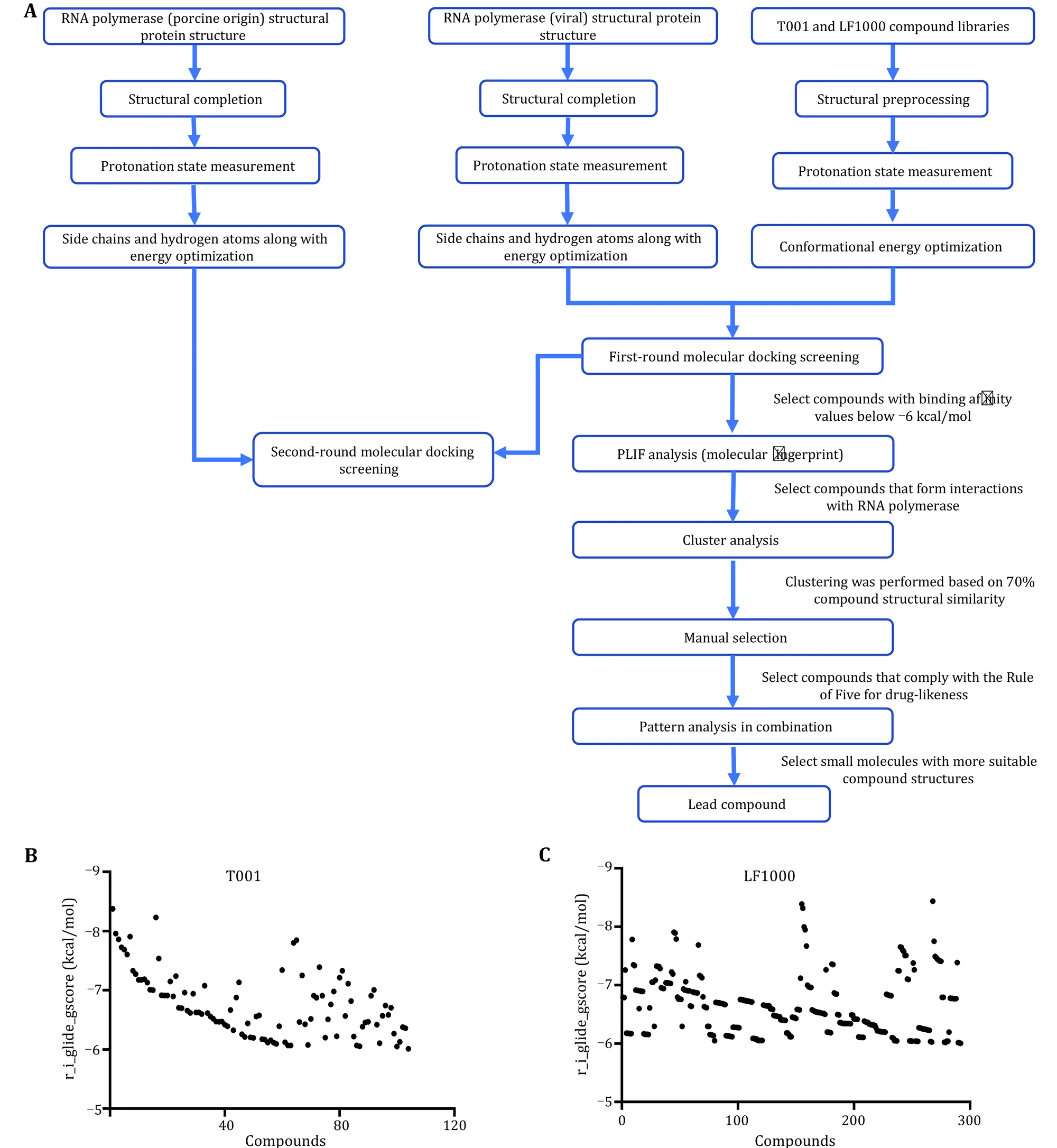
Virtual screening workflow and docking score distributions. **A** Schematic of the virtual screening pipeline, including protein preparation, ligand processing, molecular docking, and selection criteria. **B**,**C** Docking score distributions for candidate compounds from the T001 (**B**) and LF1000 (**C**) libraries

From the T001 library, 13,772 compounds initially demonstrated binding affinity to the ASFV RNAP structure, among which 367 were identified with a binding affinity for ASFV RNAP (≤−6.0 kcal/mol) that was at least two orders of magnitude stronger than for *Sus scrofa* Pol II (7NVS). Similarly, from the LF1000 library, 49,952 compounds were initially docked, with 411 compounds showing significant selectivity for ASFV RNAP over the host counterpart. Protein-ligand interaction fingerprint (PLIF) analysis indicated that the top-ranked compounds from both libraries primarily engaged the active site through specific polar and aromatic interactions — including hydrogen bonds, ionic interactions, and π-stacking — with key residues such as Asp457 (vRPB1), Arg796 (vRPB2) and Arg419 in ASFV RNAP, while exhibiting minimal interaction with equivalent residues in *Sus scrofa* Pol II (supplementary Fig. S1).

To ensure structural diversity, the candidate compounds were subjected to clustering analysis at 70% similarity using the Jarvis-Patrick algorithm. This process yielded 160 representative chemotypes from the T001 library and 325 from the LF1000 library. Subsequent application of Lipinski’s Rule of Five and visual inspection of binding modes further refined the selection to 104 compounds from T001 (with docking scores ranging from −8.38 to −6.01 kcal/mol) and 292 from LF1000 (scores from −8.44 to −6.0 kcal/mol) (Figs. 2B and 2C). Ultimately, the top 50 compounds from each library were prioritized for experimental validation.

### Cellular screening identifies K-7174 as a potent and non-cytotoxic inhibitor

The 100 selected compounds were evaluated for their ability to inhibit ASFV replication in porcine alveolar macrophages (PAMs) (supplementary Fig. S2). Cells were infected with ASFV strain CN/GS/2018-strep and treated with compounds at 10 µmol/L (Fig. 3A). Viral replication was quantified via qPCR targeting the ASFV *B646L* gene. Nine compounds exhibited inhibition rates exceeding 98% (Fig. 3B), including K-7174, CUR61414, AT9283, ML 154, Torin 2, SBI-0640756, HOE 33187, Amsacrine, and F2573-0370 (Fig. 3C). Cytotoxicity assessment revealed that K-7174 and CUR61414 were well-tolerated, with minimal impact on PAM viability (Fig. 3D). Although CUR61414 exhibited promising initial inhibition, its overall activity profile was suboptimal, characterized by a high *IC*_5__0_ (supplementary Fig. S3). Consequently, K-7174 was selected for further characterization based on its superior potency.

**Figure 3:**
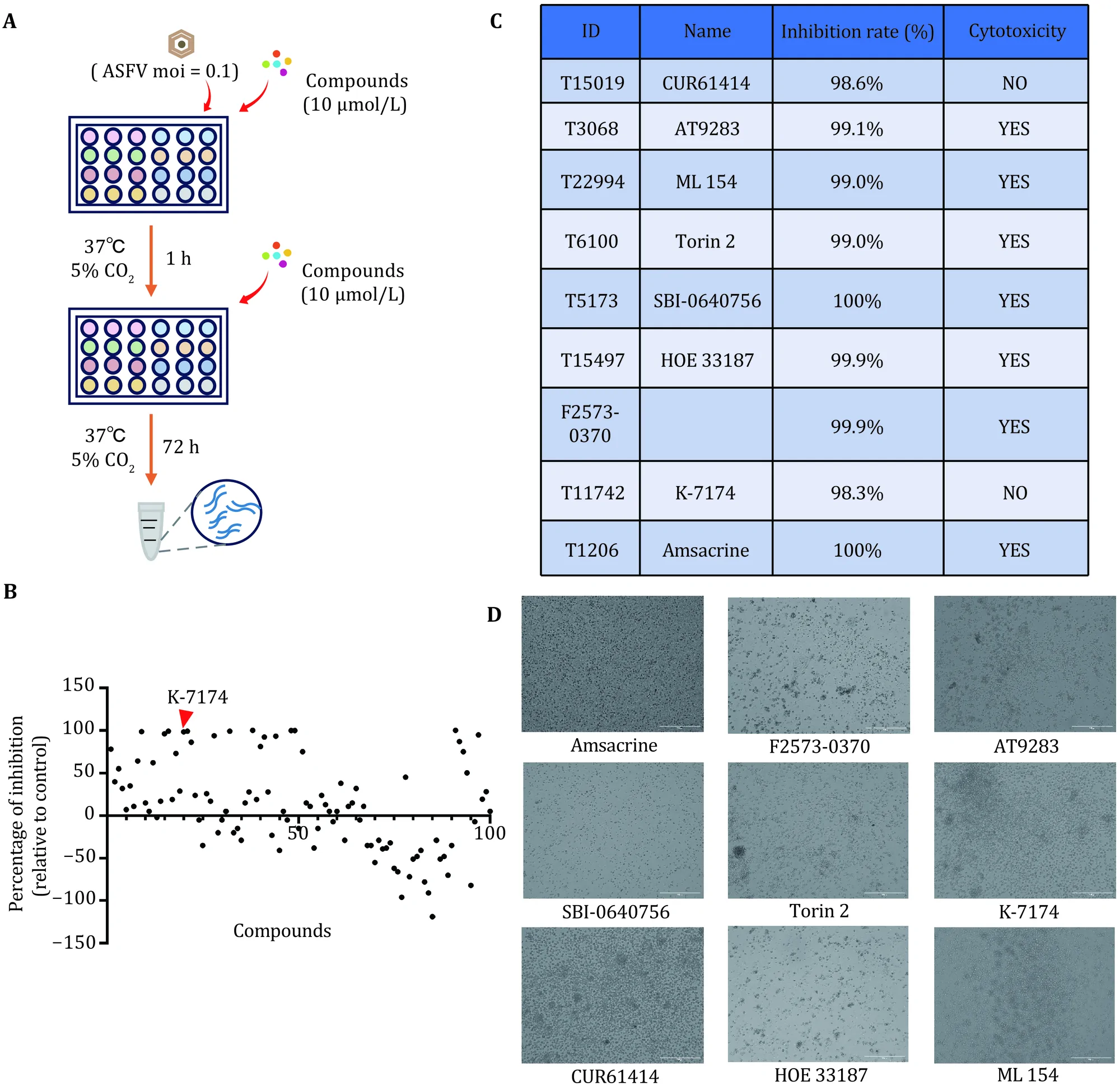
*In vitro* screening of candidate compounds for anti-ASFV activity. **A** Experimental timeline for antiviral assessment in PAMs. **B** Inhibition rates of 100 candidate compounds at 10 µmol/L. **C** Inhibition rates and cytotoxicity profiles of the top nine hits. **D** Cell viability of PAMs treated with the top nine compounds

### K-7174 exhibits dose-dependent inhibition of ASFV replication

K-7174 demonstrated significant, dose-dependent anti-ASFV activity in infected porcine alveolar macrophages (PAMs). Treatment with K-7174 (0.1−10 μmol/L) significantly reduced viral replication, as evidenced by decreased red fluorescence intensity in imaging assays (Fig. 4A) and a reduction in viral genome copies quantified by qPCR (Fig. 4B). The half-maximal inhibitory concentration (*IC*_50_) was determined to be 2.502 μmol/L (Fig. 4B). To further investigate the inhibitory effect on viral gene expression, we measured the transcript level of the late gene *B646L* (encoding p72) using RT-qPCR, which showed concentration-dependent suppression (Fig. 4C). Western blot analysis confirmed a corresponding reduction in p72 protein levels (Fig. 4D). Together, these multi-level analyses indicate that K-7174 targets multiple facets of the ASFV replication cycle.

**Figure 4:**
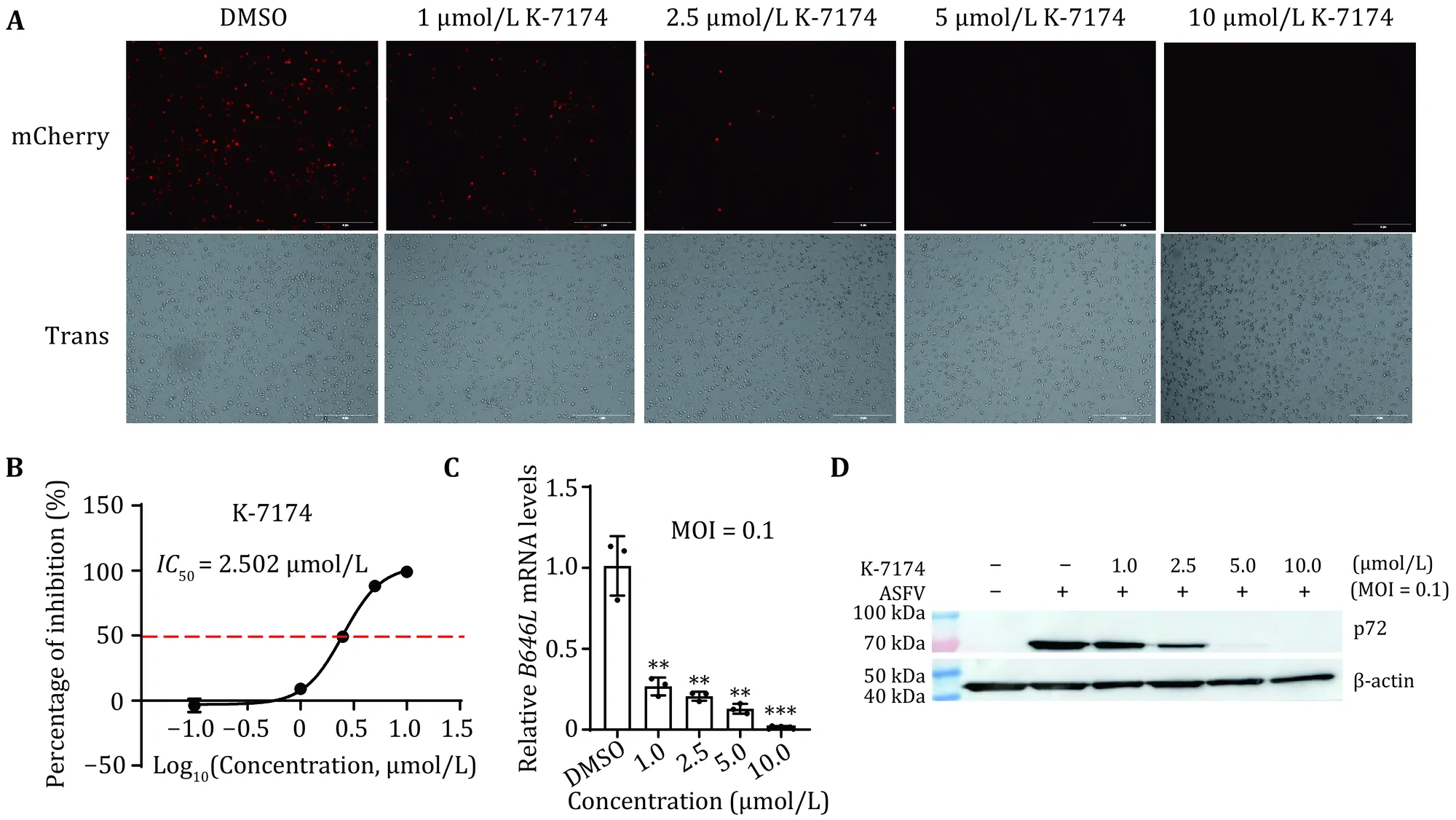
Dose-dependent inhibition of ASFV by K-7174. **A** Fluorescence imaging of ASFV-infected PAMs treated with K-7174. **B** Dose-response curve of K-7174, with an *IC*_50_ value of 2.502 µmol/L. **C** Transcript levels of the late gene *B646L* (p72) measured by RT-qPCR. **D** Protein levels of p72 analyzed by Western blot. Data are presented as mean ± SD from three independent experiments. Statistical significance was determined using GraphPad Prism 8 (**P < 0.01; ***P < 0.001)

### Molecular docking elucidates a putative mechanism of action and informs rational drug design

Molecular docking simulations provide critical structural insights into the potent inhibitory activity of K-7174 (Fig. 5A), suggesting a mechanism that can be exploited for future drug optimization. K-7174, a compound with known activity against GATA transcription factors and the proteasome, was repurposed here as a highly effective binder to the catalytic pocket of ASFV RNAP. The model predicts that K-7174 docks snugly into the Mg^2^^+^-coordinating active site, a region indispensable for nucleotide addition during RNA synthesis (Fig. 5B). Crucially, the analysis reveals that the inhibitor forms a network of high-fidelity hydrogen bonds with conserved catalytic residues: the piperazine nitrogen engages the carboxylate group of Glu1051 (2.79 Å and 3.32 Å), while the methoxy group donates a hydrogen bond to the backbone carbonyl of Ala456 (2.78 Å) (Figs. 5C and 5D). These specific interactions likely stall the polymerase by sterically hindering the entry of nucleotide triphosphates or by disrupting the precise geometry of the catalytic Mg^2^^+^ ions. The fact that K-7174 achieves such strong complementarity with the viral polymerase, while showing selectivity over the host Pol II (as evidenced by earlier screening), underscores its significant application potential as a selective antiviral agent.

**Figure 5:**
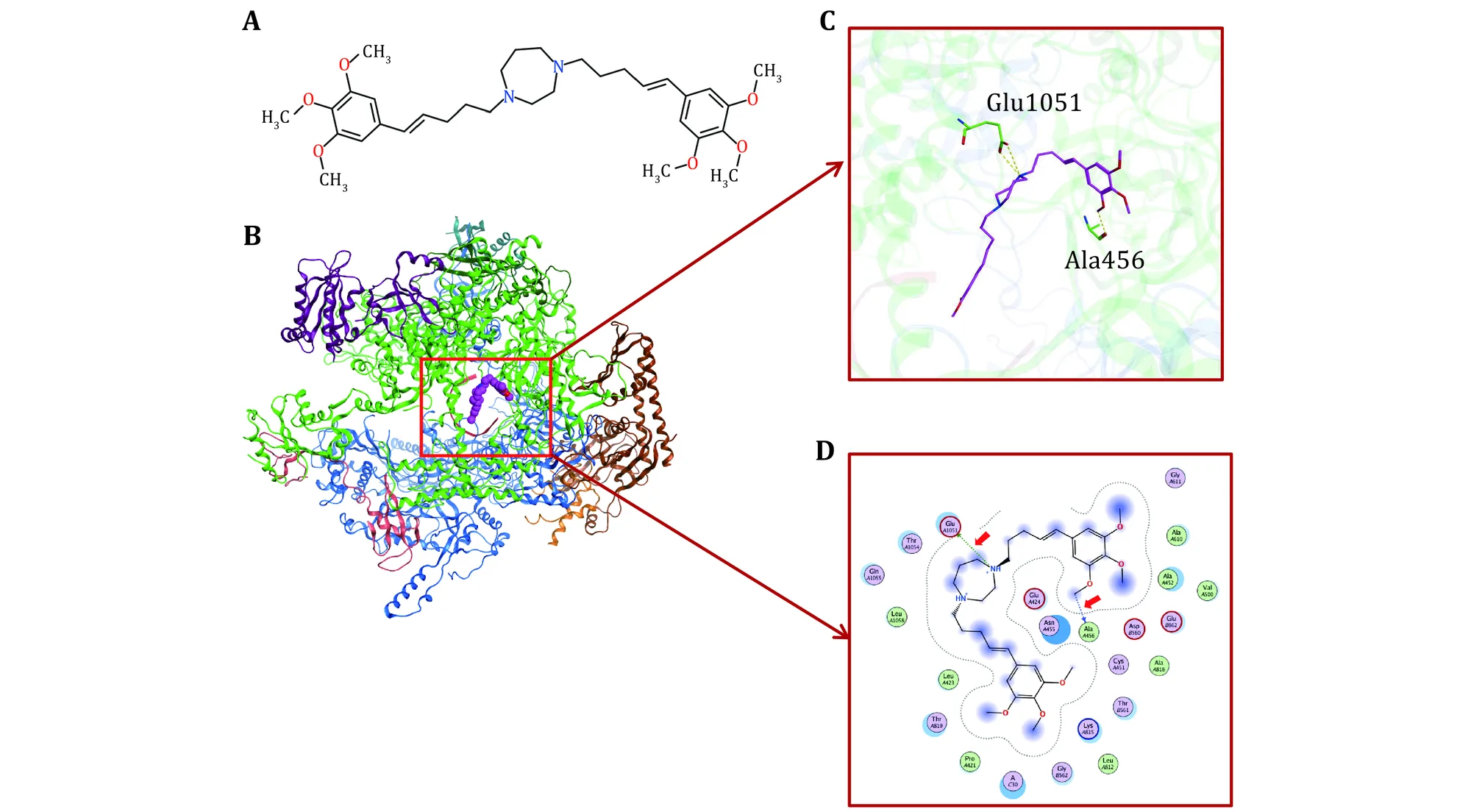
Molecular docking analysis of K-7174 bound to ASFV RNAP. **A** Chemical structure of K-7174. **B** Overall binding pose of K-7174 within the catalytic pocket. **C** Detailed 3D view of key interactions; green: carbon, red: oxygen, blue: nitrogen; yellow dashed lines: hydrogen bonds. **D** 2D interaction diagram highlighting hydrogen-bond contacts

## EXPERIMENTAL SECTION

### Cells, viruses and compounds

Primary porcine alveolar macrophages (PAMs) were isolated from 60–70-day-old specific pathogen-free (SPF) pigs. Lung lavage was performed using PBS supplemented with 2% Penicillin-Streptomycin-Gentamicin and 2% Penicillin-Streptomycin-Amphotericin B. Cells were resuspended in RPMI-1640 medium containing 10% fetal bovine serum (FBS) and maintained at 37°C under 5% CO_2_. The ASFV strains CN/GS/2018 and CN/GS/2018-strep were provided by LVRI. All compounds were purchased from TargetMol (Shanghai, China) and dissolved in DMSO or ddH_2_O to a stock concentration of 10 mmol/L.

### Structure preparation for virtual screening

The three-dimensional structures of the target proteins — the ASFV elongation complex (PDB: 8XX4) and the host *Sus scrofa* Pol II (PDB: 7NVS) — were prepared using the Protein Preparation Wizard module within the Schrödinger Maestro suite (Madhavi Sastry *et al.* 2013). The preparation workflow included: assignment of correct bond orders, addition of hydrogen atoms, optimization of protonation states at pH 7.0 using the integrated PROPKA tool and treatment of disulfide bonds. This was followed by restrained energy minimization using the OPLS4 force field to relieve atomic clashes and optimize the hydrogen-bonding network, converging the root-mean-square deviation (RMSD) of heavy atoms to within 0.3 Å. The resulting refined structures were used as receptors for docking.

### Ligand library preparation

Small molecule compounds were sourced from two distinct libraries: a curated library of known bioactive compounds (T001) and a diverse synthetic library (LF1000). All compounds were processed using the LigPrep module. This step generated possible ionization states, tautomers, and stereoisomers for each molecule at a target pH of 7.0 ± 2.0. Furthermore, to ensure adequate conformational sampling during the docking simulation, up to 32 low-energy conformers were generated for each ligand using the ConfGen tool (Greenwood *et al.* 2010; Shelley *et al.* 2007). This rigorous preparation ensured the representation of each compound’s potential bioactive form.

### Molecular docking and virtual screening workflow

The prepared receptor structures and ligand conformers were imported into the Glide module for molecular docking. The initial primary screening against the prepared ASFV RNAP structure (8XX4) was conducted using the Standard Precision (SP) scoring mode with default parameters and flexible ligand sampling (Jacobson *et al.* 2004). Post-docking, poses underwent implicit energy minimization. Compounds with a predicted binding affinity (Glide Score) of ≤−6.0 kcal/mol were classified as high-affinity hits and prioritized for counter-screening. In contrast, those with a Glide Score greater than −6.0 kcal/mol, indicative of poor binding, were discarded from further consideration (Yao *et al.* 2023).

To prioritize compounds with selectivity for the viral polymerase, all primary hits were re-docked against the prepared structure of host *Sus scrofa* Pol II (7NVS) using the identical SP protocol. Compounds demonstrating a strong binding affinity for ASFV RNAP concurrently with a significantly weaker interaction (defined as a >100-fold difference in docking score) with the host Pol II were designated as selective virtual hits.

### Interaction analysis and hit selection

The binding modes of the selective hits were subjected to detailed Protein−Ligand Interaction Fingerprint (PLIF) analysis. This method systematically quantified key interactions — including hydrogen bonds, ionic interactions, π-π stacking, and hydrophobic contacts — between the ligands and residues within the ASFV RNAP active site. Docking poses exhibiting inconsistent or non-specific interaction patterns were discarded. The final selection of candidates for *in vitro* testing was based on a holistic assessment of high docking scores, favorable and specific interaction fingerprints, and compliance with drug-like properties as evaluated by Lipinski’s Rule of Five.

### Antiviral compound screening in PAMs

PAMs were seeded in 24-well plates and infected with ASFV CN/GS/2018-strep at a multiplicity of infection (MOI) of 0.1 for 1 h at 37°C. The inoculum was removed, and the cells were washed thrice with cold PBS. Fresh maintenance medium containing the test compounds at a final concentration of 10 μmol/L was added, and the cells were incubated for 72 h. Post-incubation, the supernatants and pellets were harvested for viral DNA extraction and subsequent qPCR analysis to quantify antiviral activity, expressed as percentage inhibition compared to the DMSO-treated virus control.

### Cellular cytotoxicity assay

The cytotoxicity of compounds toward PAMs was assessed using a Cell Counting Kit-8 (CCK-8; Abbkine, Wuhan, China) according to the manufacturer’s instructions. Briefly, PAMs were seeded into 96-well plates at a density of 1 × 10^5^ cells per well and allowed to adhere for 6 h. The culture medium was then replaced with fresh medium containing serial dilutions of the test compounds (10 to 50 μmol/L). After 48 h of incubation, 10 μL of CCK-8 reagent was added to each well, and the plates were incubated for an additional 2 h. The absorbance of each well was measured at 450 nm using a microplate reader (BioTek, Vermont, USA). Cell viability was expressed as a percentage relative to the DMSO-treated control wells.

### Quantitative PCR (qPCR)

Viral genomic DNA was extracted from cell culture supernatants and pellets using a commercial DNA Viral Genome Extraction Kit (Solarbio, Beijing, China) following the manufacturer’s protocol. The highly conserved ASFV *B646L* gene (encoding the major capsid protein p72) was selected as the quantitative target. qPCR was performed using the 2× AceQ Universal U+ Probe Master Mix (Vazyme, Nanjing, China) on a QuantStudio 5 Real-Time PCR System (Thermo Fisher Scientific, Massachusetts, USA). The primer and probe sequences are listed in Table 1. A standard curve generated from serially diluted plasmids of known copy number was used to calculate the absolute viral genome copy numbers in each sample.

**Table 1:** Primer and probe sequences for qPCR

Name	Sequence (5’−3’)
*B646L*-F	CTGCTCATGGTATCAATCTTATCGA
*B646L*-R	GATACCACAAGATCAGCCGT
*B646L*-probe	FAM-CCACGGGAGGAATACCAACCCAGTG-TAMRA

### RNA extraction and reverse transcription qPCR (RT-qPCR)

Total RNA was extracted using TRIzol reagent (Vazyme) and reverse-transcribed into cDNA with HiScript II Q RT SuperMix (Vazyme) according to manufacturer protocols. cDNA was amplified with gene-specific primers (Table 2) using ChamQ Universal SYBR qPCR Master Mix (Vazyme) on a QuantStudio 5 system (Thermo Fisher Scientific). The reaction mix contained 5 µL Master Mix, 0.2 µL each of forward and reverse primers, and 4.6 µL of tenfold-diluted cDNA. Thermal cycling conditions were: 95°C for 30 s; 40 cycles of 95°C for 10 s and 60°C for 30 s. Melt curves were generated automatically. Relative mRNA levels were calculated via the 2^−∆∆CT^ method.

**Table 2:** Primer sequences for RT-qPCR

Name	Sequence (5’−3’)
*B646L*-seqF	GGCACAAGTTCGGACATGT
*B646L*-seqR	GTACTGTAACGCAGCAG
*GAPDH*-seqF	ACATGGCCTCCAAGGAGTAAGA
*GAPDH*-seqR	GATCGAGTTGGGGCTGTGACT

### Western blot

PAMs were lysed at 4°C in buffer containing 50 mmol/L HEPES (pH 7.5), 1.5 mmol/L MgCl_2_, 150 mmol/L NaCl, 0.5% NP-40, 1 mmol/L DTT, and EDTA-free protease inhibitors. Lysates were centrifuged at 10,000 r/min for 10 min, and supernatants were mixed with 5× loading buffer (Solarbio), boiled for 15 min, and separated on 10% SDS-PAGE gels. Proteins were transferred to nitrocellulose membranes, blocked with 5% skim milk in TBST for 1 h, and incubated with anti-p72 mouse monoclonal antibody (1:1000) for 2 h at room temperature. After washing, membranes were incubated with HRP-labeled goat anti-mouse IgG (1:10000; Biodragon) for 1 h. Signals were detected using an Amersham ImageQuant 800 system (Cytiva).

### Statistics

All data are expressed as the mean ± standard deviation and analyzed using GraphPad Prism 8.0 software. A value of *P* < 0.05 was considered statistically significant. (ns: not signiﬁcant, *P < 0.05, **P < 0.01, ***P < 0.001). All experiments were independently repeated at least three times.

## DISCUSSION AND CONCLUSION

African swine fever continues to inflict severe economic losses worldwide due to the lack of effective countermeasures (Sánchez-Cordón *et al.* 2018; Solikhah *et al.* 2025). In this study, we integrated high-resolution structural biology, structure-based virtual screening, and experimental virology to identify K-7174 as a novel and potent inhibitor of ASFV replication. Our multidisciplinary approach not only exemplifies the power of combining *in silico* predictions with robust biological validation to accelerate antiviral discovery but also highlights the value of targeting virus-specific transcriptional machinery (Wang *et al.* 2023).

A key highlight of this work is the successful implementation of a differential virtual screening strategy (Abduljalil and Elfiky 2022; Clyde *et al.* 2022). By leveraging the resolved structures of both ASFV RNAP (8XX4) and host *Sus scrofa* Pol II (7NVS), we specifically targeted compounds exhibiting high affinity for the viral polymerase while minimizing interaction with the host counterpart. This strategy markedly improved the selectivity and potential safety profile of the identified inhibitors, a critical consideration in antiviral development (Lu *et al.* 2023; Umemura *et al.* 2025). The subsequent experimental validation in PAMs, the primary natural target cells for ASFV, confirmed the high predictive accuracy of our computational pipeline, with nine out of one hundred candidates showing >98% inhibition.

The lead compound, K-7174, a known modulator of GATA signaling and proteasome activity (Kikuchi *et al.* 2013; Umetani *et al.* 2000), was repurposed here as a highly effective antiviral agent against ASFV. It exhibited low cytotoxicity and a dose-dependent inhibition profile with a promising *IC*_50_ of 2.502 µmol/L. More importantly, molecular docking simulations provided a plausible mechanistic basis for its activity. K-7174 is predicted to bind snugly within the catalytic pocket of ASFV RNAP, forming precise hydrogen bonds with Glu1051 (distances: 2.79 Å and 3.32 Å) and Ala456 (2.78 Å). We attempted to determine the binding affinity between K-7174 and ASFV RNAP; however, the extremely low yield and inherent instability of the purified polymerase precluded reliable measurement. Given the proximity of these residues to the Mg^2^^+^ ion cofactor essential for phosphoryl transfer during RNA synthesis, we propose that K-7174 likely acts by sterically hindering nucleotide triphosphate (NTP) entry or disrupting the optimal geometry of the catalytic metal cluster, thereby stalling the transcription elongation complex.

Beyond its immediate anti-ASFV efficacy, K-7174 possesses considerable application potential. Its well-documented pharmacokinetic and safety profiles from previous oncology and inflammation research could expedite its repurposing path (Imagawa *et al.* 2003; Kikuchi *et al.* 2013; Umetani *et al.* 2000). Furthermore, its novel binding mode within the ASFV RNAP active site offers a valuable starting point for rational drug design. Future directions should include structural biology efforts (*e*.*g*., cryo-EM of ASFV RNAP–K7174 complexes) to validate the predicted binding pose, medicinal chemistry campaigns to synthesize analogs with enhanced affinity and selectivity, and a series of *in vitro* enzymatic assays to definitively demonstrate the direct inhibition of ASFV RNAP by K-7174.

In conclusion, we report the first-in-class identification of K-7174 as a selective inhibitor of ASFV RNAP, demonstrating significant antiviral efficacy *in vitro*. This study establishes a robust and reproducible computational–experimental framework for the rapid discovery of anti-ASFV therapeutics and provides a promising chemical scaffold for further development. It also reinforces the paradigm of targeting viral transcription as a viable strategy against NCLDV.
